# Knockdown of P2Y4 ameliorates sepsis-induced acute kidney injury in mice *via* inhibiting the activation of the NF-κB/MMP8 axis

**DOI:** 10.3389/fphys.2022.953977

**Published:** 2022-08-29

**Authors:** Maojuan Wang, Fan Jiang, Lian Zhang, Juan Zhang, Hong Xie

**Affiliations:** Department of Critical Care Medicine, People’s Hospital of Deyang City, Deyang, China

**Keywords:** P2Y4, sepsis, acute kidney injury, NF-κB/MMP8 axis, mice

## Abstract

Sepsis-induced acute kidney injury (S-AKI) has emerged as a frequent and life-threatening complication in critically ill patients, which is characterized by a systematic inflammatory response and a rapid decline in kidney function. P2Y4, a member of G protein–coupled P2Y nucleotide receptor family, has been reported to serve as a crucial player in inflammatory responses during the development of neurocognitive disorder and myocardial infarction. Nonetheless, the biological role of P2Y4 in S-AKI remains largely unclear. This study aimed to decipher the biological role of P2Y4 in S-AKI and illuminate the potential mechanisms. In this study, S-AKI models were successfully established in mice *via* cecal ligation and puncture. Results showed that the kidney tissues from S-AKI mouse models exhibited a higher P2Y4 expression level than from the sham-operated group. Knockdown of P2Y4 was found to remarkably alleviate kidney damage and reduce inflammatory response in mice of S-AKI models. Moreover, P2Y4 ablation inhibited the activation of the NF-κB/MMP-8 signaling axis. Additionally, mechanistic studies revealed that rescuing MMP-8 reversed the alleviating effects of P2Y4 knockdown against renal cell damage. Collectively, our findings indicate that P2Y4 knockdown ameliorated S-AKI in mice *via* inhibiting the activation of the NF-κB/MMP-8 axis and that P2Y4 may represent a novel therapeutic target for S-AKI patients.

## Introduction

Sepsis, a complex clinical syndrome, is characterized by systematic inflammatory responses and multiple organ dysfunctions (Alobaidi et al., 2015; Peerapornratana et al., 2019; Poston and Koyner, 2019). Acute kidney injury (AKI) is a common and life-threatening complication of sepsis in hospitalized and critically ill patients (Kellum et al., 2019; Petejova et al., 2020). It is well documented that AKI occurs in nearly 50% of septic patients, leading to a mortality rate of up to 40% (Hoste et al., 2018; Kalantari and Rosner, 2021). Sepsis-induced AKI (S-AKI) is often featured by a rapid decline in kidney function and a severe inflammatory response (Li et al., 2020; Wang et al., 2021). The NF-κB signaling axis has been widely recognized to be closely associated with inflammatory response in tissue injury. It is well documented that NF-κB is usually trapped in the cytoplasm under the normal physiological condition and its nuclear translocation would promote the downstream pro-inflammatory cytokine production when tissue injury occurs (Cai et al., 2015; Poon et al., 2015; Ding et al., 2018). In spite of the fact that some advances have been made in treating S-AKI in recent decades, the prognosis of patients remains pretty poor. Hence, it is in urgent demand to further decipher the etiology and mechanism underlying septic AKI, thereby developing novel and efficient therapeutic strategies.

It is widely accepted that abnormal release and accumulation of purine and pyrimidine nucleotides in extracellular matrix is considered as a critical biological event in organ damages and inflammatory disorders (Xu et al., 2018; Giuliani et al., 2019; Vallon et al., 2020). The purinergic type 2 receptor family, namely, P2 receptor family, belongs to the superfamily of G protein–coupled receptors (Zhang et al., 2020; Li et al., 2021; Lu et al., 2021). Mounting evidence has demonstrated that P2 receptors are closely associated with the pathological development of inflammatory diseases, including acute kidney injury, acute lung damage, and acute liver injury (Howarth et al., 2015; Menzies et al., 2017; Monaghan et al., 2021). P2Y4, a receptor of uridine triphosphate (UTP), is a novel member of P2 family of receptors (Zhang et al., 2019). It is noteworthy that P2Y4 has been recently revealed to serve a crucial role during the development of traumatic diseases, such as neuronal damage and myocardial infarction (Horckmans et al., 2015; Zhou et al., 2019). Nevertheless, the biological role of P2Y4 in S-AKI remains largely unknown.

In this study, we aimed to explore the potential role of P2Y4 in mouse models of S-AKI and elucidate the potential molecular mechanisms involved. Herein, cecal ligation and puncture (CLP) was used to establish S-AKI mouse models. Notably, P2Y4 expression patterns were characterized in renal tissues in sham-operated mice and CLP-treated mice. Moreover, effects of P2Y4 on kidney damage and inflammatory cytokine production were evaluated in S-AKI mouse models. Additionally, functional studies were conducted *via in vitro* damage models of renal tubular epithelial cells. Collectively, this study may offer new insights into understanding the role of P2Y4 in S-AKI and provide some evidence for P2Y4 as a potential target in S-AKI treatment.

## Materials and methods

### Clinical sample collection and analysis

Human blood samples were collected from 20 healthy volunteers and 25 S-AKI patients at Deyang People’s Hospital. This study involving human participants was approved by the Research Ethics Committee of Deyang People’s Hospital (approval number: IRBDHP201908056; chairman: Biao Zhang; date: 10 August 2019). And all the patients gave written informed consent.

P2Y4 mRNA expression levels were examined using the quantitative TaqMan PCR method in serum samples. The specific primers and probes were synthesized by Thermo Fisher (Rockford, IL, United States), and their sequences were listed as follows: P2Y4 forward primer, 5′-CCT​TAA​CGC​CCC​AAC​CCT-3′; P2Y4 reverse primer, ACA​GCA​CAT​ACA​AGG​TGT​CT; P2Y4 probe, 5′-CCT​CCG​ACC​CTG​GGA​TGC​AAC-3’; and GAPDH forward primer, 5′-ATC​GTG​GAA​GGA​CTC​ATG​ACC-3′; GAPDH reverse primer, 5′-TGC​CAG​TGA​GCT​TCC​CGT​TC-3′; GAPDH probe, 5′-CCA​TCA​CGC​CAC​AGT​TTC​CCG​GAG-3’. PCR analysis was performed on the ABI 7500 PCR system (Applied Biosystems, Foster City, CA, United States). GAPDH was used as an internal control. P2Y4 mRNA expression levels in the serum samples were determined *via* the 2^-△△Ct^ method.

### Animals

Male C57/BL6 (8 weeks old) mice were purchased from the Shanghai Laboratory Animal Center (Shanghai, China). All the mice were housed in separated plastic cages under a 12-h light/dark cycle in an air-conditioned facility with free access to water and standard chow.

### Adeno-associated virus vector construction

Recombinant adeno-associated virus vectors carrying specific short hairpin RNA targeting P2Y4 (shP2Y4) or scramble negative control short hairpin RNA (shNC) were constructed by the Shanghai GenePharma Company (Shanghai, China).

### Animal experiments

Animal experiments were conducted in Deyang People’s Hospital (Deyang, China). All the animal experiments were approved by the Committee of Animal Use and Care of Deyang People’s Hospital (approval number: DPH202003091201; chairman: Gang Mai; date: 9 March 2020). Cecal ligation and puncture (CLP) was used to establish mouse models. In the first animal experiment, sixteen mice were classified into two groups (*n* =8 in each group): the sham-operated group and the CLP treatment group. In the second animal experiment, thirty-two mice were classified into four groups (*n* =8 in each group): the sham-operated group, the CLP treatment group, the CLP + shNC group, and the CLP + shP2Y4 group. For the CLP + shNC group and the CLP + shP2Y4 group, 200 μl of adeno-associated vectors carrying shNC and shP2Y4 (2 × 10^9^ PFU/ml) was injected into tail vein of mice at half an hour after CLP modeling, respectively. Mice were killed at 24 h after the experimental treatment *via* carbon dioxide inhalation. In this study, no death was observed within 24 h post-treatment. Renal tissues and blood samples were then collected for further analysis.

The protocol for CLP model establishment was described as follows: After anesthesia, the abdomens of mice were incised to exteriorize the cecum. The cecum was ligated 1 cm from the cecal tip using the 4–0 silk thread and perforated using the 21 G needle. A small amount of feces were then gently squeezed from the perforated site. The cecum was repositioned to the abdominal cavity. The abdomen was closed in two layers, and 1 ml of normal saline was injected subcutaneously for recovery.

### Cell culture and cell transfection

Human renal proximal tubular epithelial cell line HK-2 was purchased from the Shanghai Cell Bank of Chinese Academy of Sciences (Shanghai, China). HK-2 cells were cultured in DMEM containing 10% fetal bovine serum (Invitrogen, Carlsbad, CA, China) at 37°C in a CO_2_ incubator. Lipofectamine 2000 (Invitrogen) was used to carry out cell transfection according to the manufacturer’s protocols. Short hairpin RNA specifically targeting P2Y4 (shP2Y4) and scramble negative control short hairpin RNA (shNC) were designed and constructed by the Shanghai GeneChem Co., Ltd. (Shanghai, China). Transfection efficiency was evaluated at 48 h post-transfection *via* Western blotting analysis.

### *In vitro* cell damage model

*In vitro* cell damage models were established in HK-2 cells *via* the lipopolysaccharide (LPS) stimulus method. To be specific, HK-2 cells were incubated with 1 μg/ml LPS (Sigma, St. Louis, MO, United States) for 24 h.

### Histopathological examination and kidney injury scoring

Histopathological examination was performed by hematoxylin–eosin (H&E) staining of renal tissues. In brief, renal tissues were fixed in 4% paraformaldehyde overnight, followed by dehydration, paraffin-embedding, slicing, deparaffinization, and staining according to the routine method. A kidney injury score was evaluated based on pathological manifestations (tubular atrophy or dilatation, loss of brush border, vacuolization, epithelial cell shedding, and denuded tubular basement membrane) and was scored as follows: 0, normal; 1, less than 10%; 2, 10%–25%; 3, 25%–50%; 4, 50%–75%; and 5, 75%–100%.

### Enzyme-linked immunosorbent assay

ELISA kits (Nanjing Jiancheng Biotech, Nanjing, China) were used to determine the concentrations of blood urea nitrogen (BUN) and serum creatinine (SCr) in the serum, uKIM-1 in the urine, and TNF-α, IL-6, and MCP-1 in the serum and renal tissues according to the manufacturer’s instructions. Each experiment was performed in triplicates.

### Quantitative real-time PCR analysis

Total RNA was isolated using TRIzol reagent (Thermo Fisher) in line with the manufacturer’s instructions. After quantifying isolated total RNA, cDNA was synthesized using High Capacity cDNA Reverse Transcription Kit containing RNase inhibitor (Thermo Fisher). PCR analysis was conducted using the SYBR Select Master Mix (Applied Biosystems). Specific primers were designed and synthesized by the Shanghai Sangon Biotech Company (Shanghai, China). The specific primers were listed as follows: for GAPDH, 5′-ACT​CAA​GAT​TGT​CAG​CAA​TGC-3’ (forward) and 5′-ATG​AGC​CCT​TCC​ACA​ATG​CCA-3’ (reverse); and for P2Y4, 5′-CCT​TAC​CCT​CCA​CCC​TCT​TC-3’ (forward) and 5′-CAA​GGA​GTC​TGC​ACT​GGT​CA-3’ (reverse). The relative expression level of P2Y4 mRNA was calculated using the 2^−△△Ct^ method with GAPDH as an internal control.

### Western blotting analysis

Total proteins were extracted using RIPA lysis buffer (Sigma). 20 μg of proteins was separated *via* SDS-PAGE and was then transferred onto PVDF membranes. After being blocked with 5% nonfat milk, membranes were incubated with primary antibodies overnight at 4°C. Primary antibodies anti-P2Y4 (catalog no. ab180718), anti-GAPDH (catalog no. ab9485), anti-MMP-8 (catalog no. ab53017), anti-p65 (catalog no. ab32536), and anti-phospho-p65 (Ser536) (catalog no. 76302) were purchased from Abcam (Cambridge, MA, United States). Protein bands were then visualized using ECL detection reagent (Pierce, Rockford, IL, United States) following incubation with an HRP-labeled secondary antibody for 1 h. Quantification of protein bands was carried out using the ImageJ software.

### Immunohistochemical staining analysis

Immunohistochemical staining analysis was performed to visualize the distribution of P2Y4 in renal tissues. In brief, the deparaffinized slices were incubated with primary antibody anti-P2Y4 (Abcam, catalog no. ab180718) overnight at 4°C, followed by incubation with biotinylated secondary antibody for 30 min. Subsequently, positive P2Y4 staining was observed under a light microscope (BX52, Olympus, Tokyo, Japan).

### Cell viability analysis

Cell viability was determined *via* MTT assay. Briefly, HK-2 cells were seeded into 96-well plates at a concentration of 5 × 10^4^ cells per well in 100 μl of culture medium and were incubated for 24 h. Subsequently, 10 μl of MTT solution (0.5 mg/ml) was added to each well and was incubated for another 4 h. After discarding MTT solution, DMSO was added to dissolve formazan. Finally, the absorbance was measured at a wavelength of 570 nm.

### Statistical analysis

Results were presented as mean ± standardized deviation. Statistical analysis was conducted using the SPSS software. Student’s *t*-test was used to evaluate the statistical significance of differences between the two groups. One-way ANOVA followed by the Dunnett multiple comparison test was applied to assess the statistical significance of differences among three or more groups. A *p*-value less than 0.05 was considered statistically significant.

## Results

### Increased P2Y4 expression is observed in renal tissues of acute kidney injury mice

**FIGURE 1:**
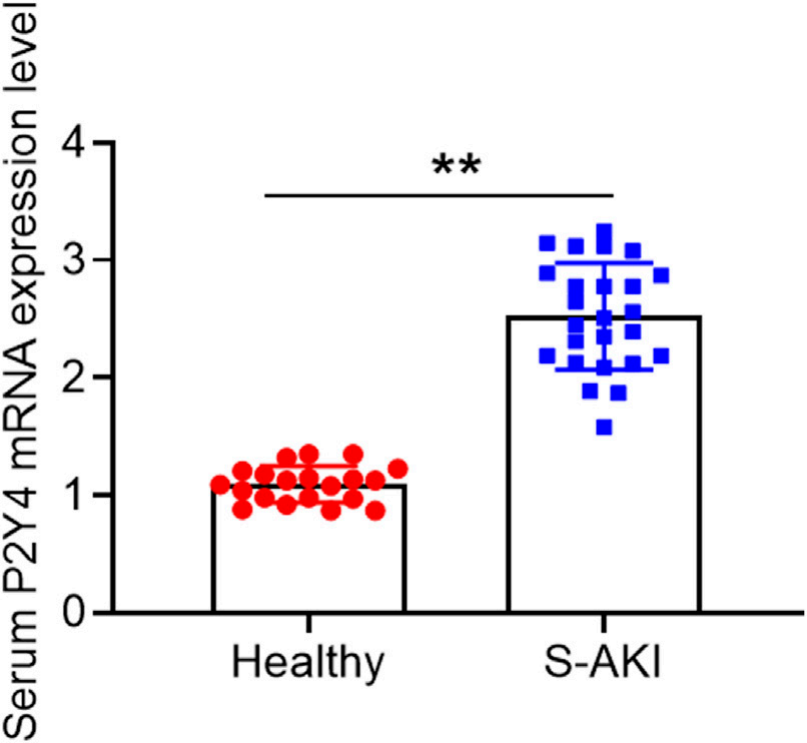
P2Y4 mRNA expression levels in human serum samples. P2Y4 mRNA expression levels were examined using the quantitative TaqMan PCR method in serum samples of 20 healthy volunteers and 25 S-AKI patients.

**FIGURE 2:**
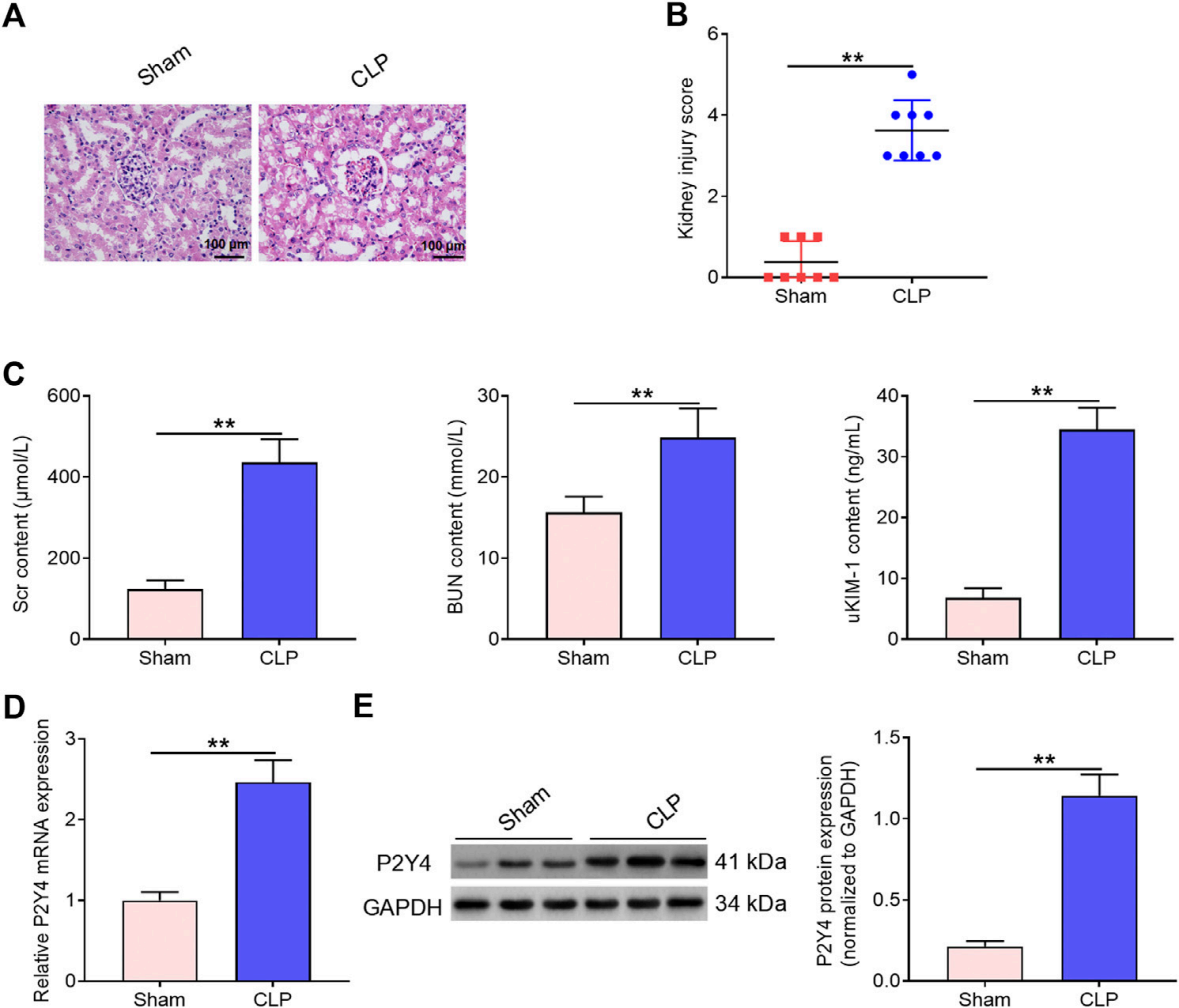
Increased P2Y4 expression is observed in renal tissues of S-AKI mice. **(A)** H&E staining was used to evaluate histopathological changes in renal tissues of CLP-treated mice (*n* = 6). **(B)** Kidney injury score was evaluated in sham-operated mice and CLP-treated mice, respectively. **(C)** Concentrations of serum biomarkers (BUN and Scr) and urinary biomarker (uKIM-1) were determined by ELISA in sham-operated mice and CLP-treated mice, respectively. **(D)** P2Y4 mRNA levels were examined by qRT-PCR in renal tissues of CLP-treated and sham-operated mice, respectively. **(E)** P2Y4 protein expression levels were determined by Western blotting in renal tissues of CLP-treated and sham-operated mice, respectively. **p < 0.01. S-AKI, sepsis-induced acute kidney injury; H&E staining, hematoxylin and eosin staining; SCr, serum creatinine; BUN, blood urea nitrogen; uKIM-1, urinary kidney injury molecule-1; CLP, cecal ligation and puncture; ELISA, enzyme-linked immunosorbent assay.

In human blood samples, serum samples of S-AKI patients displayed higher P2Y4 mRNA expression levels than those of healthy volunteers (Figure 1). Moreover, the detailed results of P2Y4 mRNA expression levels in human serum samples were also provided in Supplementary Table S1. To investigate the potential biological role of P2Y4 in sepsis-induced AKI, we firstly established AKI models in mice using the CLP method. As shown in Figure 2A, kidney tissues in the sham-operated group appeared to be normal, whereas significant pathological changes were observed in kidney tissues of S-AKI mice, including vacuolization, epithelial cell shedding, and denuded tubular basement membrane. Meanwhile, higher kidney injury scores were found in mice of S-AKI models than in the sham-operated group (Figure 2B). Moreover, ELISA analyses showed a remarkable increase in the concentrations of key biomarkers for kidney damage, namely, SCr, BUN, and uKIM-1, in the CLP treatment group (Figure 2C, *p* < 0.01). Subsequently, P2Y4 mRNA expression and protein expression in kidney tissues were examined by qRT-PCR and Western blotting, respectively. As presented in Figures 2D,E, kidney tissues from S-AKI mice exhibited higher P2Y4 mRNA and protein expression levels than those from the sham-operated group (*p* < 0.01). Taken together, these results suggest that P2Y4 expression is significantly increased in renal tissues of S-AKI mice.

### P2Y4 knockdown alleviates kidney damage in mice of sepsis-induced acute kidney injury models

**FIGURE 3:**
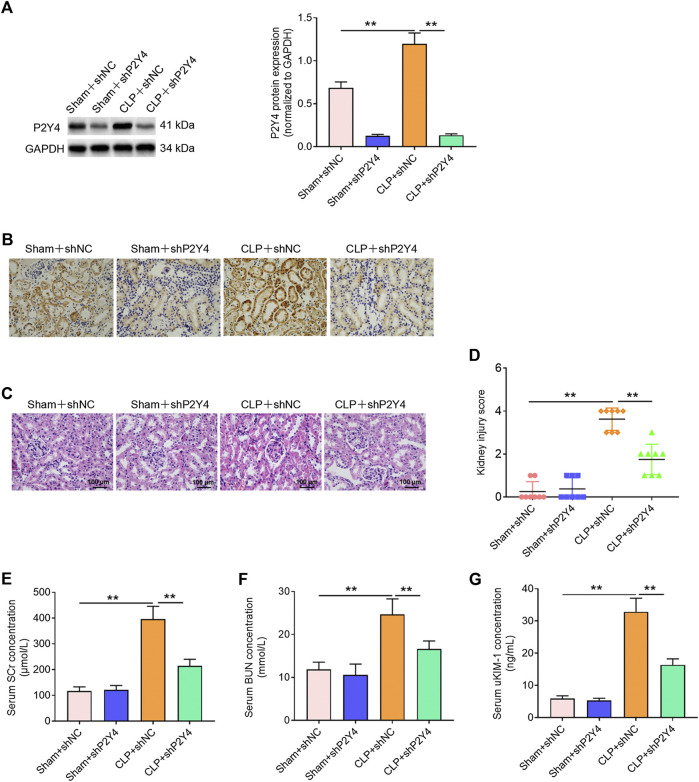
P2Y4 knockdown alleviates kidney damage in mice of S-AKI models. **(A)** Western blotting was used to evaluate P2Y4 protein expression in renal tissues of mice following the tail intravenous injection of adeno-associated vectors carrying shNC or shP2Y4. **(B)** Immunohistochemical staining was performed to visualize the distribution of P2Y4 in renal tissues of mice. **(C)** H&E staining was used to visualize histological manifestation in renal tissues from different treatment groups. **(D)** Kidney injury scores were assessed in renal tissues of mice from different groups. **(E–G)** Concentrations of serum biomarkers (BUN and Scr) and urinary biomarker (uKIM-1) were determined *via* ELISA in different groups. **p < 0.01. S-AKI, sepsis-induced acute kidney injury; H&E staining, hematoxylin and eosin staining; CLP, cecal ligation and puncture; SCr, serum creatinine; BUN, blood urea nitrogen; uKIM-1, urinary kidney injury molecule-1; shNC, negative control short hairpin RNA; shP2Y4, specific short hairpin RNA targeting P2Y4; ELISA, enzyme-linked immunosorbent assay.

To further explore the biological role of P2Y4 in S-AKI, the effects of P2Y4 knockdown on kidney damage were analyzed *via* tail intravenous injection of adeno-associated virus vector carrying specific shP2Y4. Western blotting was used to evaluate the knockdown efficiency of P2Y4 in kidney tissues (Figure 3A). Moreover, the temporal analyses of adenovirus-induced P2Y4 knockdown efficiency demonstrated that a significant knockdown of P2Y4 expression was earliest found at 20 h post-injection and that P2Y4 expression was still effectively downregulated at 24 h post-injection when the mice were killed for assessing the possible protective effect (Supplementary Figure S1). Immunohistochemical staining showed that P2Y4 protein was distributed in renal tubules rather than in other segments of the kidney (Figure 3B). Furthermore, it was observed that CLP treatment induced the upregulation of P2Y4 expression in renal tubules and that adenovirus injection reduced P2Y4 expression in renal tubules (Figure 3B). As exhibited in Figures 3C,D, P2Y4 knockdown significantly relieved histopathological changes and decreased kidney injury scores in renal tissues of S-AKI mice in comparison with negative control treatment (*p* < 0.01). Moreover, ELISA analyses showed that P2Y4 ablation notably reversed the increases in SCr, BUN, and uKIM-1 induced by cecal ligation and puncture treatment (Figures 3E–G, *p* < 0.01). Collectively, these results indicate that P2Y4 knockdown ameliorates kidney damage in mice of S-AKI models.

### P2Y4 ablation ameliorates inflammatory responses in mice of sepsis-induced acute kidney injury models

**FIGURE 4:**
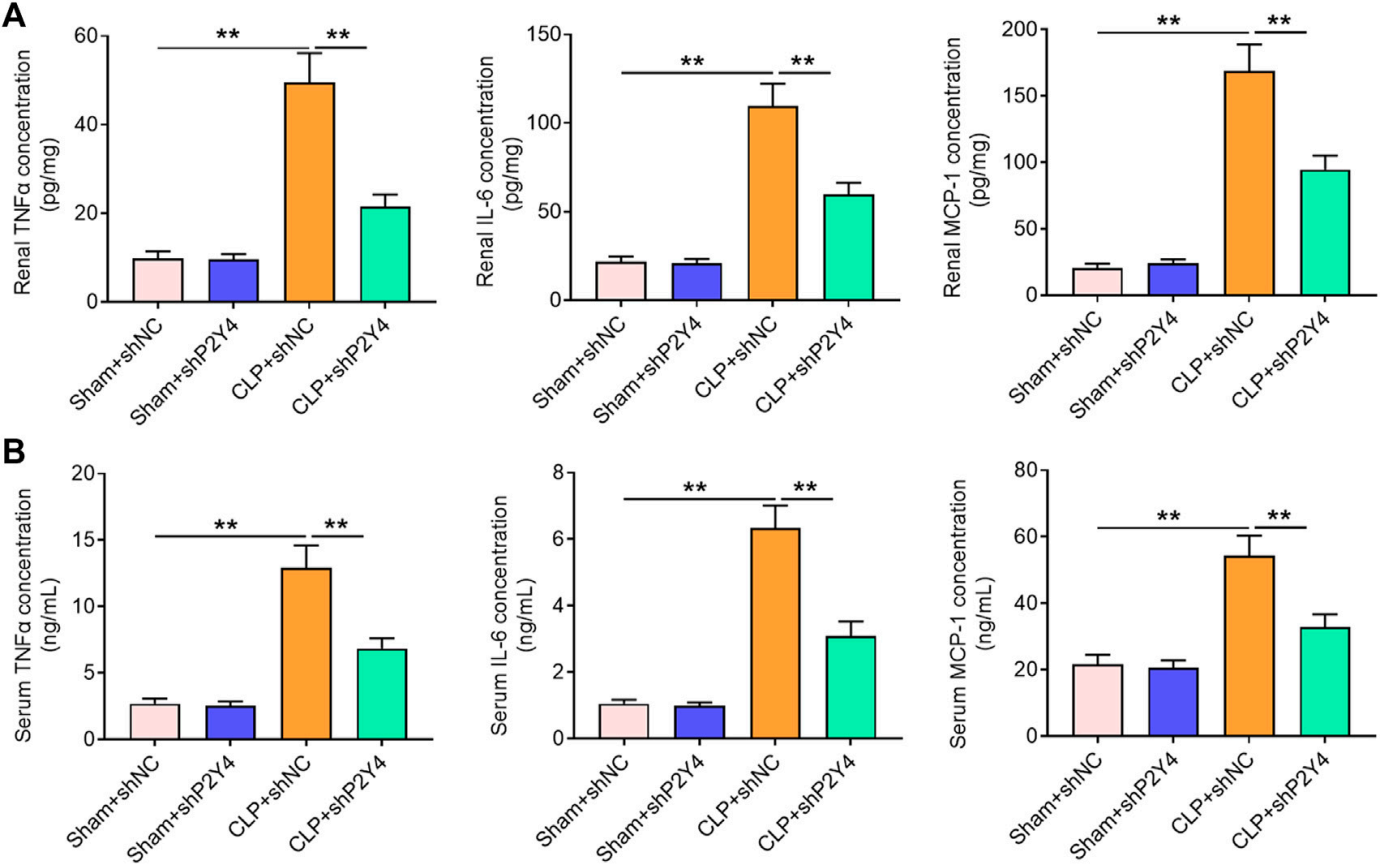
P2Y4 ablation ameliorates inflammatory responses in mice of S-AKI models. Concentrations of key inflammatory cytokines TNF-α, IL-6, and MCP-1 were determined by ELISA in **(A)** renal tissues and **(B)** serum of mice from different groups. **p < 0.01. S-AKI, sepsis-induced acute kidney injury; CLP, cecal ligation and puncture; TNF-α, tumor necrosis factor-α; IL-6, interleukin-6; MCP-1, monocyte chemoattractant protein-1; ELISA, enzyme-linked immunosorbent assay; shNC, negative control short hairpin RNA; shP2Y4, specific short hairpin RNA targeting P2Y4.

It is widely acknowledged that a severe inflammatory response is a crucial hallmark of S-AKI. Herein, the effects of P2Y4 knockdown on inflammatory cytokines TNF-α, IL-8, and MCP-1 were assessed in serum and renal tissues of AKI mice. As displayed in Figure 4A, P2Y4 knockdown abolished the notable increases in concentrations of TNF-α, IL-8, and MCP-1 in renal tissues of S-AKI mice (*p* < 0.01). Moreover, P2Y4 ablation was also observed to dramatically reverse the increases in serum TNF-α, IL-8, and MCP-1 levels of S-AKI mice (Figure 4B, *p* < 0.01). To sum up, these results demonstrate that P2Y4 ablation relieves inflammatory responses in S-AKI mice.

### P2Y4 knockdown inhibits the activation of the NF-κB/MMP-8 axis in both sepsis-induced acute kidney injury mouse models and *in vitro* cell damage models

**FIGURE 5:**
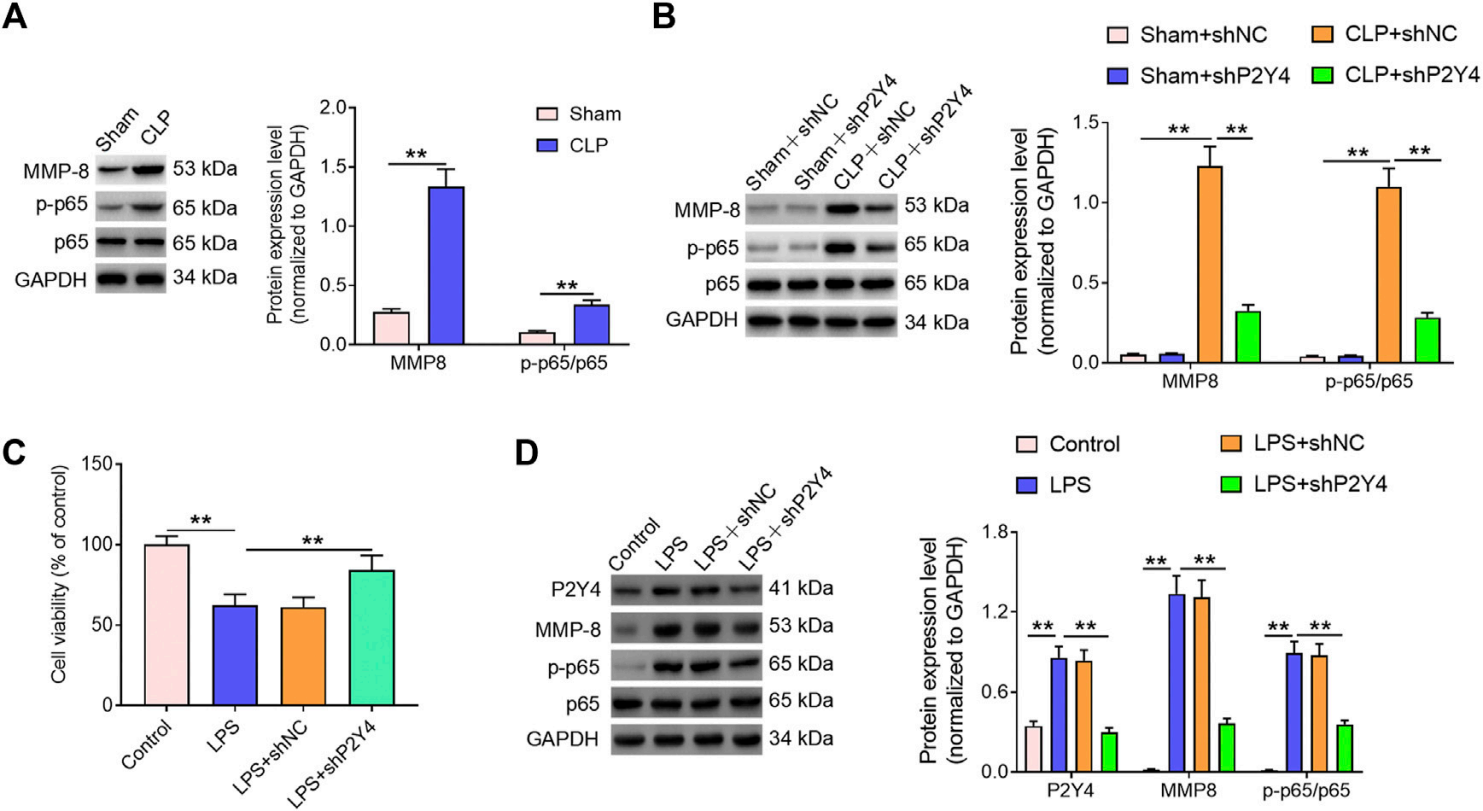
P2Y4 knockdown inhibits the activation of the NF-κB/MMP-8 axis in both S-AKI mouse models and *in vitro* cell damage models. **(A)** Expression levels of p65, p-p65, and MMP-8 proteins were detected using Western blotting in renal tissues of CLP-treated and sham-operated mice, respectively. **(B)** Expression levels of p65, p-p65, and MMP-8 proteins were determined *via* Western blotting in renal tissues of mice from different groups. **(C)** Cell viability was analyzed in different treatment groups. **(D)** Expression levels of p65, p-p65, and MMP-8 proteins were analyzed *via* Western blotting in HK-2 cells exposed to different treatments. **p < 0.01. S-AKI, sepsis-induced acute kidney injury; CLP, cecal ligation and puncture; NF-κB/MMP-8, nuclear factor kappa B/matrix metalloproteinase 8; p-p65, phosphorylated p65.

Mounting evidence has revealed that the NF-κB/MMP-8 axis acts as a key role in inflammatory disorders (Jeong et al., 2015; Ong et al., 2015; Mu et al., 2017; Lee et al., 2018). In order to explore whether the NF-κB/MMP-8 signaling pathway was involved in the development of S-AKI, Western blotting was then performed. As shown in Figure 5A, higher NF-κB p-p65 and MMP-8 expression levels were found in renal tissues of S-AKI mice than in those of the sham-operated group (*p* < 0.01). Notably, P2Y4 knockdown significantly reversed the activation of the NF-κB/MMP-8 axis in renal tissues of S-AKI mice (Figure 5B, *p* < 0.01). In addition, *in vitro* cell damage model was constructed *via* the LPS stimulus (1 μg/ml) in HK-2 cells, a human proximal renal tubular epithelial cell line. As exhibited in Figure 5C, the LPS stimulus led to a significant decrease in HK-2 cell viability (*p* < 0.01). In consistent with the findings in S-AKI mouse models, the LPS stimulus significantly increased P2Y4 protein expression levels and activated the NF-κB/MMP-8 axis in HK-2 cell damage models compared with the control group (Figure 5D, *p* < 0.01). Moreover, P2Y4 knockdown notably reversed the decrease in cell viability triggered by LPS treatment (Figure 5C) and dramatically inhibited the activation of the NF-κB/MMP-8 axis in LPS-treated HK-2 cells (Figure 5D). These findings suggest that P2Y4 knockdown suppresses the activation of the NF-κB/MMP-8 axis in both S-AKI mouse models and *in vitro* cell damage models.

### Rescuing MMP-8 expression reverses the alleviating effects of P2Y4 knockdown against renal cell damage

**FIGURE 6:**
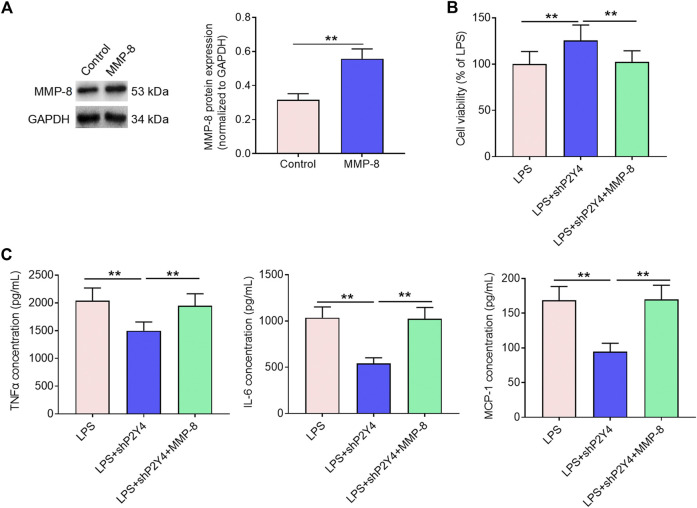
Rescuing MMP-8 reverses the alleviating effects of P2Y4 knockdown against renal cell damage. **(A)** MMP-8 protein expression was evaluated *via* Western blotting in shP2Y4-treated HK-2 cells after transfection of MMP-8 expression vector. **(B)** Cell viability was determined by MTT assays in different groups. **(C)** TNF-α, IL-6, and MCP-1 concentrations in different groups were examined by ELISA. **p < 0.01. MMP-8, matrix metalloproteinase 8; LPS, lipopolysaccharide; TNF-α, tumor necrosis factor-α; IL-6, interleukin-6; MCP-1, monocyte chemoattractant protein-1; ELISA, enzyme-linked immunosorbent assay; shP2Y4, specific short hairpin RNA targeting P2Y4.

Based on the results mentioned above, subsequent mechanistic studies were conducted in HK-2 human proximal tubular epithelial cells. In order to evaluate whether the NF-κB/MMP-8 axis mediates the protective effects of P2Y4 ablation against renal cell damage, we rescued MMP-8 expression in shP2Y4-treated HK-2 cells *via* transfection of MMP-8 expression vectors (Figure 6A). Cell viability analysis demonstrated that MMP-8 upregulation significantly destroyed the protective effects of P2Y4 knockdown against LPS-induced HK-2 renal cell injury (Figure 6B). Besides, MMP-8 upregulation also reversed the increases in concentrations of key inflammatory cytokines TNF-α, IL-8, and MCP-1 in the culture supernatant (Figure 6C). Collectively, these results suggest that the NF-κB/MMP-8 axis mediates the protective effects of P2Y4 knockdown against renal cell damage.

**FIGURE 7:**
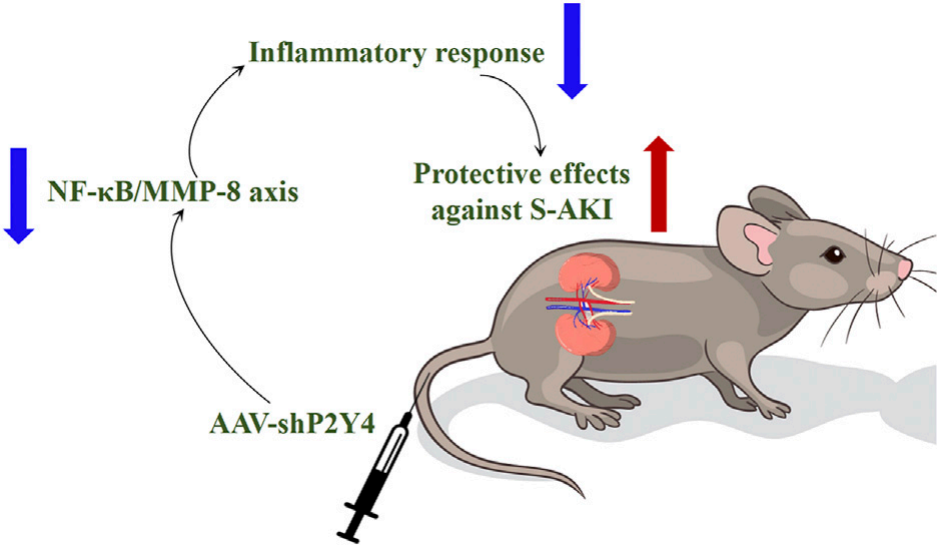
Proposed schematic diagram summarizes the research finding that P2Y4 knockdown ameliorates S-AKI in mouse models by repressing the activation of the NF-κB/MMP-8 axis. S-AKI, sepsis-induced acute kidney injury; NF-κB/MMP-8, nuclear factor kappa B/matrix metalloproteinase 8; AAV-shP2Y4, adeno-associated vector carrying specific short hairpin RNA targeting P2Y4.

Considering all the findings in this study, it is proposed that P2Y4 knockdown alleviates S-AKI partly through repressing the activation of the NF-κB/MMP-8 axis as depicted in the schematic diagram (Figure 7).

## Discussion

In recent decades, S-AKI has aroused widespread attention owing to its high morbidity and mortality (Manrique-Caballero et al., 2021). It is widely acknowledged that sepsis represents the first cause of AKI occurrence, imposing tremendous health burdens on patients worldwide (Wang et al., 2018). Despite some progress in the therapy of S-AKI, the treatment outcomes are still unsatisfied. Thus, it is of vital importance to unveil the pathogenesis of S-AKI, thereby boosting the development of efficient therapeutic options. It is noteworthy that excessive release and hyper-accumulation of nucleotides is a pivotal event in organ injury, including renal damage (Idzko et al., 2014; Kishore et al., 2018). P2Y4, a novel member of the P2 nucleotide receptor family, has been uncovered to exert pro-inflammatory effects in traumatic diseases (Horckmans et al., 2015; Zhou et al., 2019). Nonetheless, the role of P2Y4 in S-AKI remains poorly understood.

In the present study, we successfully constructed S-AKI mouse models *via* the CLP method, as evidenced by the examination of histopathological changes and the detection of injury biomarkers. Subsequently, the expression profiles of P2Y4 were determined in renal tissues from sham-operated mice and S-AKI mice *via* qPCR and Western blotting analyses. Interestingly, it was discovered that P2Y4 expression was significantly elevated in S-AKI mice in comparison with that in the sham-operated group. In order to investigate the potential role of P2Y4 in S-AKI, we then evaluate the effects of P2Y4 knockdown in mouse models. Intriguingly, P2Y4 ablation was observed to significantly attenuate renal damage in murine models of S-AKI. It is well documented that excessive inflammatory response is a crucial hallmark of S-AKI (Gómez and Kellum, 2016; Bellomo et al., 2017; Perazella, 2019). It was found that P2Y4 knockdown reversed the remarkable increase in TNF-α, IL-6, and MCP-1 in the serum and renal tissues in S-AKI mice. These results indicated that P2Y4 knockdown could exert protective effects against CLP-induced AKI in mouse models.

In order to illuminate the potential molecular mechanisms by which P2Y4 ablation exerted its protective effects against CLP-induced AKI, subsequent mechanistic research was carried out. Mounting evidence has proposed that the aberrant activation of the NF-κB pathway and an excessive expression of its downstream effector MMP-8 could result in a severe inflammatory response (Jeong et al., 2015; Mu et al., 2017; Lee et al., 2018). Furthermore, recent studies have reported that the abnormal activation of the NF-κB/MMP-8 axis is largely responsible for inflammatory disorders (Ong et al., 2015; Zuo et al., 2020).

In the present study, it was discovered that the NF-κB/MMP-8 signaling pathway was significantly activated in renal tissues of S-AKI mice in comparison with that of sham-operated mice and that P2Y4 knockdown notably reversed the activation of the NF-κB/MMP-8 signaling pathway triggered by CLP treatment. In addition, *in vitro* damage models were established in human renal epithelial cell line HK-2 *via* the LPS stimulus. In line with the observations *in vivo*, it was noticed that P2Y4 was highly expressed in LPS-treated HK-2 cells and P2Y4 ablation dramatically reversed the activation of the NF-κB/MMP-8 signaling axis in HK-2 cell damage models. With the purpose of further clarifying the protective mechanism, we rescued MMP-8 expression in shP2Y4-treated cell damage models. It was discovered that rescuing MMP-8 reversed the alleviating effects of P2Y4 knockdown against the LPS stimulus-induced renal cell damage. Therefore, on the basis of all the observations in the current study, it is proposed that the NF-κB/MMP-8 axis mediates the protective effects of P2Y4 ablation against S-AKI and that P2Y4 knockdown attenuates S-AKI in mouse models through suppressing the activation of the NF-κB/MMP-8 axis. Nevertheless, there are some limitations existing in this study that we must acknowledge. Further studies should be conducted to explore the effects of P2Y4 knockdown on the survival rate of CLP-induced S-AKI mice at different time points after the experimental treatment, which would enrich our knowledge about the biological role of P2Y4 in S-AKI and lay the foundation for P2Y4 as a candidate therapeutic target. Besides, more in-depth and comprehensive mechanistic studies should be carried out by means of advanced omics technologies in our future research, such as transcriptomics analysis and proteomics analysis. Moreover, recent reports have demonstrated that these omics analyses would facilitate the comprehensive revelation of inflammatory disorders, including S-AKI (Chen et al., 2020; Akıncılar et al., 2021; Yang et al., 2022). Taken together, deeper studies are still required to further illuminate the molecular mechanisms in the future research.

In summary, the present study demonstrated for the first time that P2Y4 expression was significantly increased in both S-AKI mouse models and *in vitro* renal cell damage models. Moreover, functional studies showed that P2Y4 knockdown ameliorated damage in mouse models and *in vitro* cell models. Furthermore, mechanistic research unveiled that the NF-κB/MMP-8 axis mediated the protective effects of P2Y4 ablation against renal damage. Overall, our study may offer some new insights into clarifying the role of P2Y4 in S-AKI and provide some bases for P2Y4 as a promising target for S-AKI patients.

## Data Availability

The raw data supporting the conclusions of this article will be made available by the authors, without undue reservation.
