# Supplementary material for: Knockdown of P2Y4 ameliorates sepsis-induced acute kidney injury in mice via inhibiting the activation of the NF-κB/MMP8 axis
Source: Front Physiol. 2022 Aug 29;13:953977. doi: 10.3389/fphys.2022.953977 (PMC9467379; doi:10.3389/fphys.2022.953977)
Supplement: Supplementary file 1 [file Table1.DOCX]

**Table S1. P2Y4 mRNA expression levels in human serum samples**

| No. | P2Y4 mRNA expression levels | |
| --- | --- | --- |
|  | Healthy volunteers (n=20) | S-AKI patients (n=25) |
| 1 | 1.35 | 2.51 |
| 2 | 0.98 | 2.78 |
| 3 | 1.35 | 3.12 |
| 4 | 1.21 | 2.12 |
| 5 | 1.09 | 3.12 |
| 6 | 0.98 | 2.87 |
| 7 | 1.32 | 3.15 |
| 8 | 1.14 | 1.89 |
| 9 | 1.04 | 2.13 |
| 10 | 1.23 | 2.45 |
| 11 | 1.13 | 2.19 |
| 12 | 1.18 | 2.56 |
| 13 | 1.08 | 2.31 |
| 14 | 0.87 | 2.09 |
| 15 | 0.88 | 1.87 |
| 16 | 0.92 | 2.39 |
| 17 | 0.97 | 1.58 |
| 18 | 0.87 | 2.35 |
| 19 | 1.15 | 2.65 |
| 20 | 1.13 | 2.78 |
| 21 |  | 2.89 |
| 22 |  | 3.25 |
| 23 |  | 2.19 |
| 24 |  | 2.78 |
| 25 |  | 3.08 |
