# Supplementary material for: Knockdown of P2Y4 ameliorates sepsis-induced acute kidney injury in mice via inhibiting the activation of the NF-κB/MMP8 axis
Source: Front Physiol. 2022 Aug 29;13:953977. doi: 10.3389/fphys.2022.953977 (PMC9467379; doi:10.3389/fphys.2022.953977)
Supplement: Supplementary file 2 [file Table2.DOCX]

**Figure S1**

**
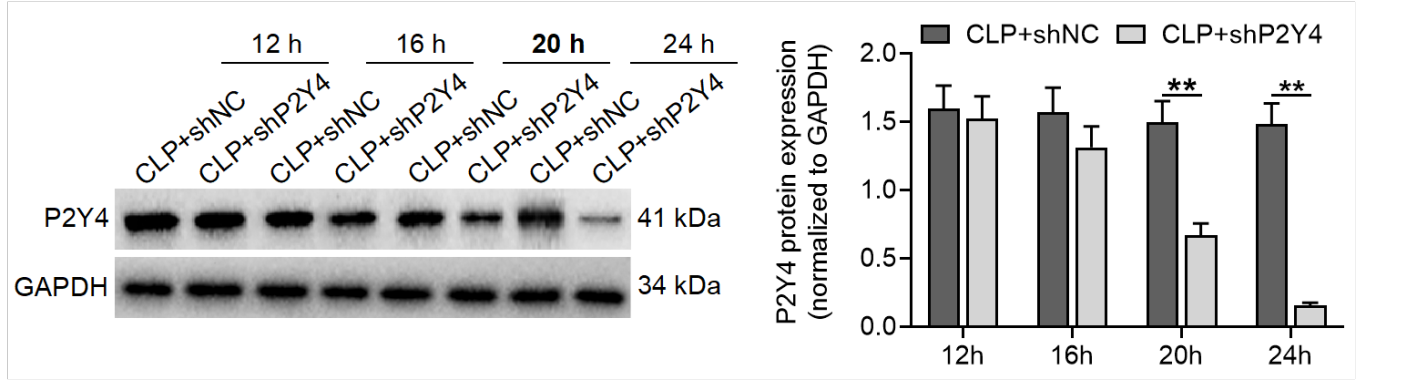
**

**Figure S1**. Temporal analyses of adenovirus-induced P2Y4 knockdown efficiency *in vivo*. P2Y4 protein expression in the renal tissue was evaluated at 12h, 16 h, 20 h and 24 h post-injection, respectively.
