# Supplementary material for: Knockdown of P2Y4 ameliorates sepsis-induced acute kidney injury in mice via inhibiting the activation of the NF-κB/MMP8 axis
Source: Front Physiol. 2022 Aug 29;13:953977. doi: 10.3389/fphys.2022.953977 (PMC9467379; doi:10.3389/fphys.2022.953977)

Supplementary files for original WB bands

Figure 1E

Band for GAPDH

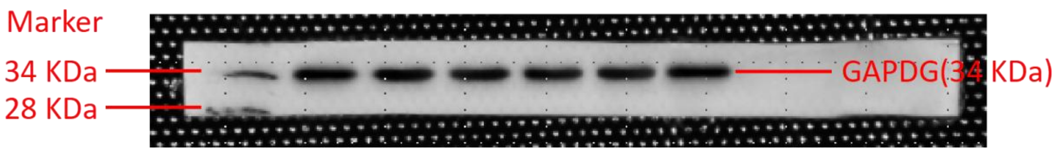

Band for P2Y4

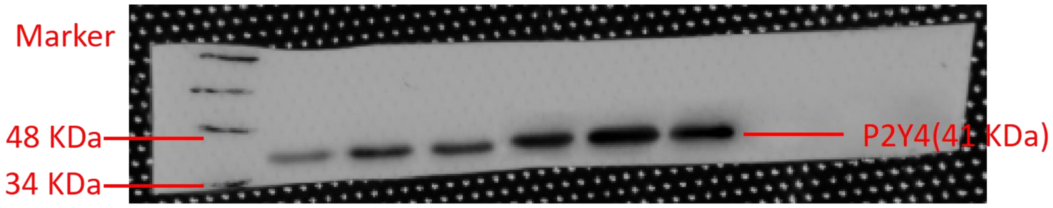

Figure 2A

Band for GAPDH

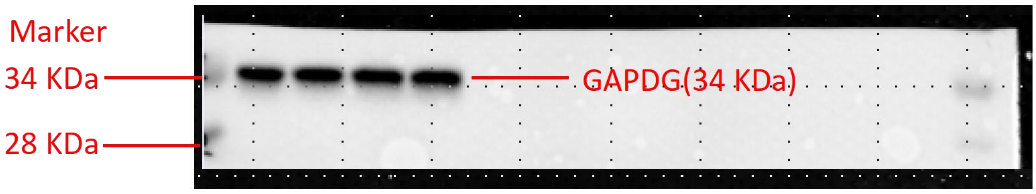

Band for P2Y4

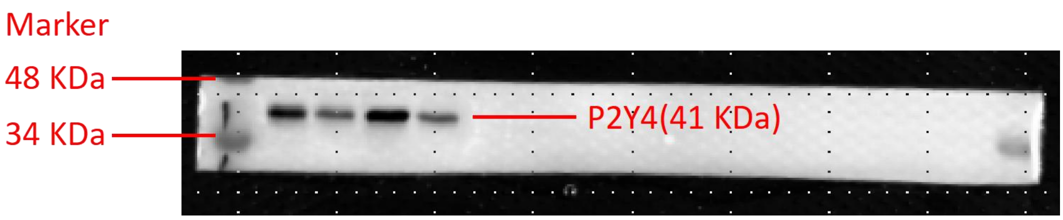

Figure 4A

Band for GAPDH

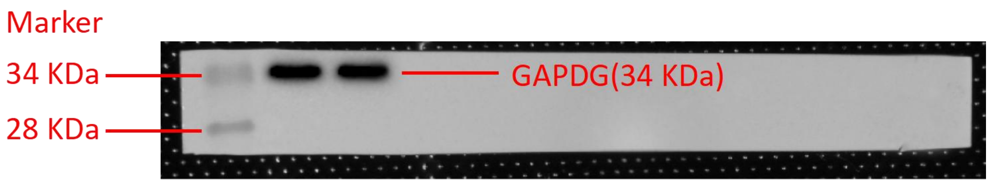

Band for MMP-8

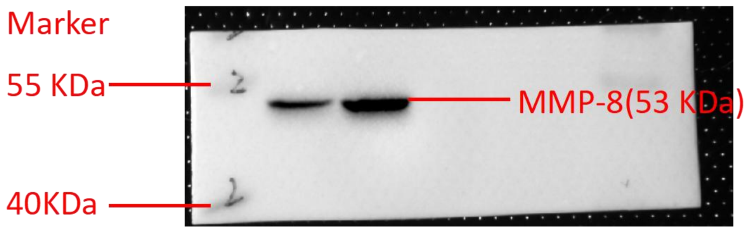

Band for p65

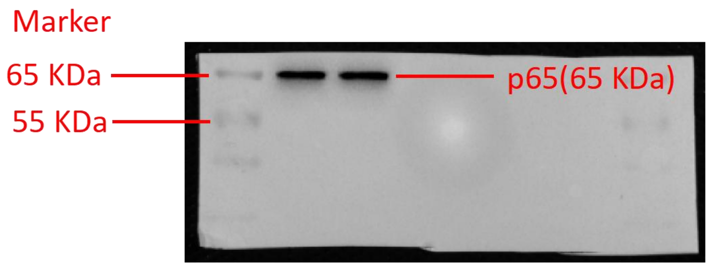

Band for p-p65

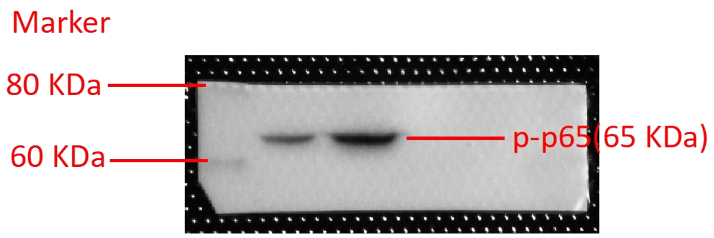

Figure 4B

Band for GAPDH

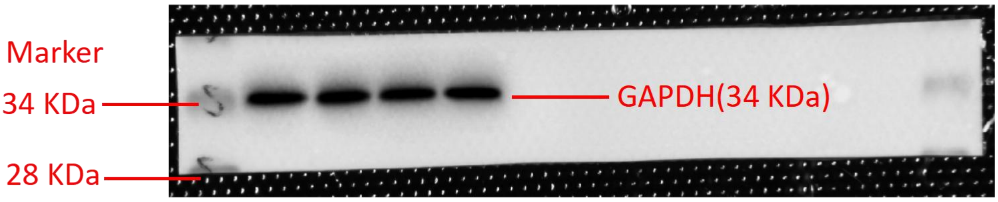

Band for MMP-8

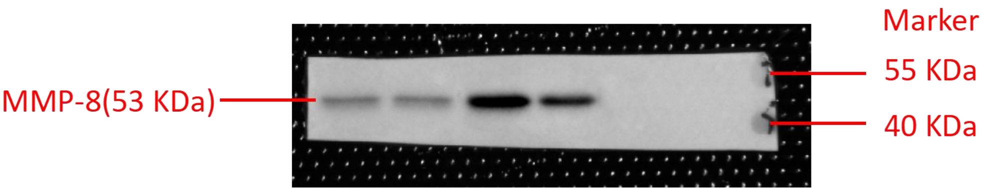

Band for p65

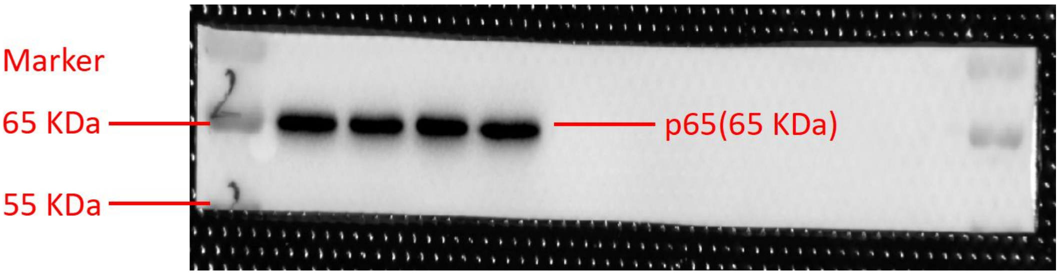

Band for p-p65

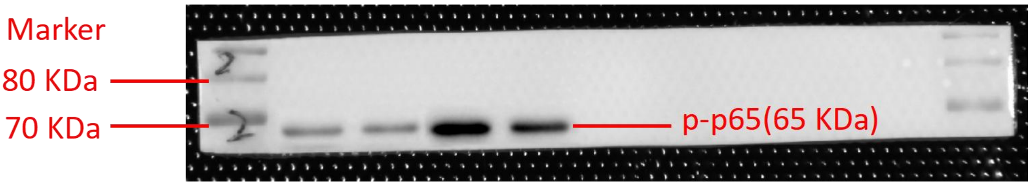

Figure 4D

Band for GAPDH

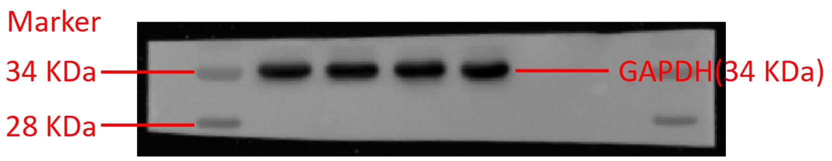

Band for MMP8

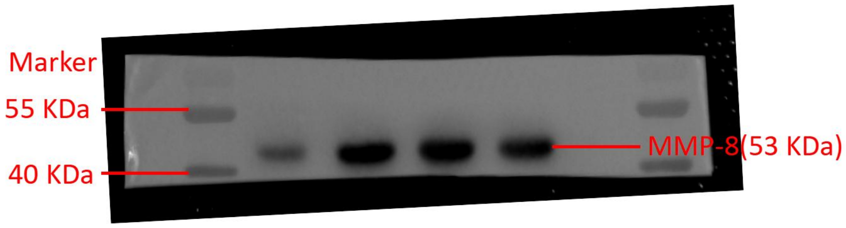

Band for P2Y4

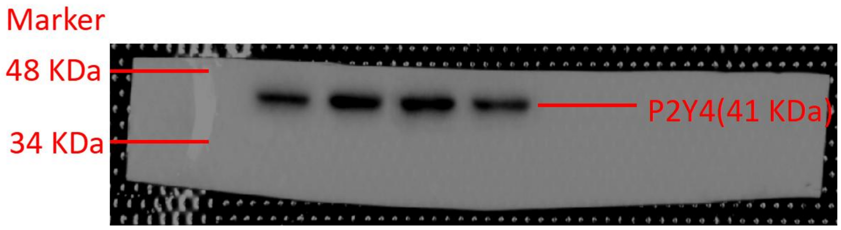

Band for p65

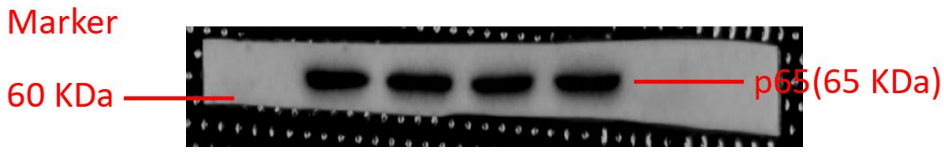

Band for p-p65

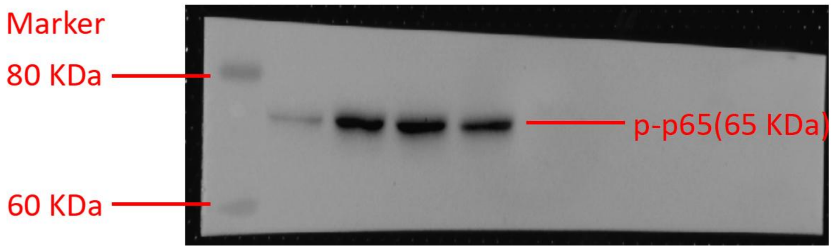

Figure 5A

Band for GAPDH

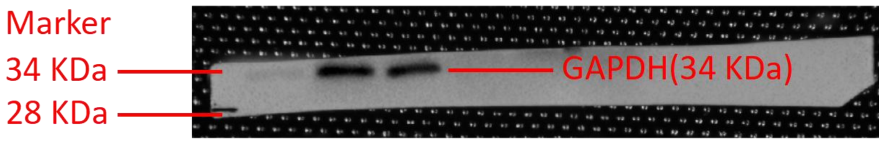

Band for MMP-8

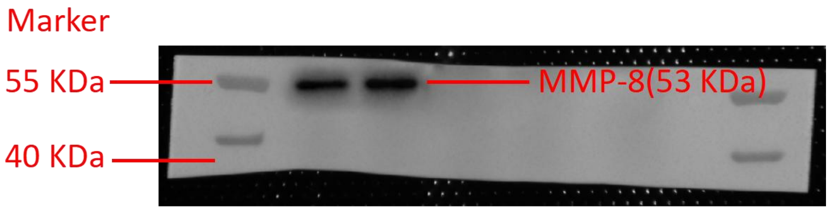

Supplement: Supplementary file 3 [file DataSheet1.PDF]
